# Airborne dust and microorganisms from hay produced on intensively and extensively managed grasslands in Switzerland

**DOI:** 10.1016/j.vas.2026.100706

**Published:** 2026-05-20

**Authors:** Jan Kocher, Christelle Mossu, Jasmin Wandel, Georg Kaim, Beat Reidy, Conny Herholz

**Affiliations:** a School of Agricultural, Forest and Food Sciences HAFL, Bern University of Applied Sciences, Laenggasse 85 3052 Zollikofen, Switzerland; b Institute for Optimization and Data Analysis, Bern University of Applied Sciences, Biel, Switzerland; c Laboratory of Molecular Analyses, PCR-Lab Services GbR 77855 Achern, Germany

**Keywords:** Barn or field drying, Odor, Respirable particles, Standardized sampling, qPCR

## Abstract

Hay is a key component of equine nutrition, supplying fibre essential for gastrointestinal function and natural chewing behavior. However, it is also a major source of respirable dust and airborne microorganisms in stables and thus a relevant risk factor for equine asthma. This study quantified PM₂.₅ concentrations and airborne microbial loads from hay derived from intensively managed (HI; *n* = 11) and extensively managed (HE; *n* = 7) grasslands and evaluated the effects of hay type and drying method. Eighteen samples were characterized for dry matter, functional, structural composition, and sensory quality. Respirable dust was measured using a standardized vibration system with a laser dust sensor, and airborne microorganisms were sampled and quantified by qPCR. Fifteen variables were analyzed using principal component analysis followed by hierarchical clustering, identifying four clusters. The cluster comprising HI, barn-dried hay showed the lowest airborne contamination (PM₂.₅: 115 µg/m³; total aerobic, mesophilic bacteria: 10,288 CFU/m³; molds: 2238 CFU/m³; yeasts: 1163 CFU/m³; actinomycetes: 500 CFU/m³) and the highest sensory scores. In contrast, clusters dominated by HE hay, particularly field-dried samples, exhibited markedly higher PM₂.₅ (up to 310 µg/m³) and microbial load (up to 54,122 CFU/m³). While drying method alone had limited influence on PM₂.₅, intensive grassland management combined with barn drying consistently reduced dust and microbial exposure. Selecting such hay may reduce airborne contaminants in stables and should be considered in preventive and therapeutic strategies for equine respiratory health.

## Highlights

• Standardized PM₂.₅ and bioaerosol sampling.
• qPCR quantified bacteria, molds, yeasts, actinomycetes.
• Dust monitoring, qPCR and clustering identified four hay groups.
• Hay type and drying method affected dust, microbes, quality.
• Experimental framework for airborne exposure in equine respiratory health.

## 1 Introduction

In the wild, horses feed slowly and continuously, spending 10 to 15 h per day grazing (Ellis, 2010; Harris et al., 2017). In domestic settings, this natural feeding pattern must be reproduced through a fibre-rich diet in which forage plays a central role in covering nutritional needs, stimulating prolonged chewing activity, and supporting digestive health and overall well-being (Cavallini et al., 2022; Coenen & Vervuert, 2010; Wyss & Strickler, 2011). Since pasture alone may not meet these requirements, particularly in winter or when access is restricted, the use of preserved forage is indispensable (Cavallini et al., 2022; Jugovic et al., 2023). However, hay is one of the main sources of dust and airborne microorganisms in stables, contributing substantially to poor air quality (Clements & Pirie, 2007; Ivester et al., 2014). High concentrations of fungal spores, bacteria, and organic debris released from forage act as persistent irritants to the equine respiratory tract (Claussen & Hessel, 2017; Fritz, 2017; Mańkowska & Witkowska, 2024).

While reference values exist for assessing the microbiological quality of hay itself (Coenen & Vervuert, 2020; Kamphues, 2013), considerably less information is available on the quantity of microorganisms emitted into the air by hay of different qualities, particularly in the breathing zone of horses with respiratory disorders such as equine asthma (Bouverat et al., 2025a).

Respirable dust is defined as the fraction of airborne particles capable of reaching the peripheral airways. Approximately 80% of living airborne components are attached to dust particles, where they play a major role in the pathogenesis of equine asthma (Pirie et al., 2003). In this context, particulate matter (PM) is commonly classified according to its aerodynamic diameter, most frequently into PM₁₀ (<10 µm) and PM₂.₅ (<2.5 µm) (Fritz, 2017; Garlipp et al., 2010; Ivester et al., 2014). However, a more detailed, health-related classification considers the inhalable fraction (<100 µm), which comprises all particles entering the respiratory tract, including the extrathoracic fraction (10–100 µm) depositing in the upper airways, the thoracic fraction (PM₁₀, <10 µm) reaching the trachea and bronchi, the respirable fraction (PM₄, <4 µm) which represents particles capable of reaching the gas-exchange (alveolar) region.

These size-dependent fractions are closely linked to deposition patterns within the equine respiratory system. Particles between 10 and 100 µm are primarily trapped in the upper airways, whereas particles measuring 5–10 µm may reach the trachea and bronchi (Hessel et al., 2009; Hwang et al., 2020). The smallest particles, particularly those between 1 and 5 µm, are considered most harmful because they can penetrate deep into the most sensitive regions of the lungs and induce chronic inflammatory conditions such as equine asthma (Hessel et al., 2009; Hwang et al., 2020; Ivester et al., 2014; Millerick-May et al., 2013). Notably, while PM₁₀ and PM₂.₅ are defined based on aerodynamic diameter, inhalable, thoracic, and respirable fractions are health-related categories derived from human data, and deposition efficiencies for these fractions have not yet been fully established in horses (Bouverat et al., 2025a).

The sources of bioaerosols in stables are multiple and closely related to equine activity and environmental conditions. The horse itself actively contributes to this load through hair loss, skin cell shedding, and the emission of germs through natural behaviors such as coughing or sneezing (Mańkowska & Witkowska, 2024; Saastamoinen et al., 2015; Szabo, 2008; Wathes et al., 1983). Additional sources include organic materials such as bedding, hay and concentrates, which release dust and microbes during handling or decomposition (Art et al., 2002; Fritz, 2017; Garlipp et al., 2010; Hwang et al., 2020; Saastamoinen et al., 2015; Szabo, 2008). Factors such as humidity, temperature, ventilation, and cleaning practices (notably sweeping) further influence airborne microbial concentrations (Fleming et al., 2009; Harris et al., 2017; Labie et al., 2019; Mańkowska & Witkowska, 2024).

Microorganisms present in stable air include various bacteria, fungi (molds and yeasts) and actinomycetes (Fritz, 2017). Knowledge of the dry matter (DM) content of the forage is essential, since too much moisture provides ideal conditions for mold proliferation (Kamphues, 2013; Resch et al., 2014). Actinomycetes develop primarily in overly moist or moldy hay and indicate advanced deterioration of the material, often accompanied by heat production inside the bale (Dutkiewicz et al., 1994; Fritz, 2017; VDLUFA, 2017). Their ability to form mycotoxins makes them particularly relevant for equine respiratory health (Dutkiewicz et al., 1994; Müller, 2012; VDLUFA, 2017).

To date, no official reference values exist for microbial concentrations in stable air, complicating the evaluation of microbial load (Gołofit-Szymczak & Górny, 2010; Witkowska et al., 2012). However, thresholds exist for feedstuffs, established by the VDLUFA (2017), which proposes a classification method for the microbiological quality of forage based on the number of molds, yeasts, and bacteria (Nesic et al., 2020). This approach distinguishes four quality classes, ranging from I (desirable quality) to IV (feed unfit for animal consumption), according to microorganism concentration relative to a set reference value (Nesic et al., 2020).

In Switzerland, hay is produced from intensively managed, permanent, or temporary grasslands as well as from extensively managed grasslands. The latter typically yield species-rich forage, which is frequently used in horse feeding. When harvested at a more mature stage to increase fibre proportion, such forage tends to be lower in sugar and protein Tüscher et al. (2021). Species-rich grasslands often include a wide variety of herbaceous species (Bodas et al., 2008; Fraisse et al., 2007; Ineichen et al., 2019), which may influence drying characteristics and, consequently, hygienic quality.

The aim of this study was to quantify the release of airborne dust and microorganisms from hay produced from intensively managed grassland (HI) and extensively managed grassland (HE) using standardized methods. It was hypothesized that hay from extensively managed grassland (HE) would receive lower scores in sensory evaluation and exhibit higher dust and microbial counts than hay from intensively managed systems (HI).

## 2 Material and methods

### 2.1 Hay samples

A total of 18 hay samples were collected for this study (Table 1). All samples were produced by farmers and represented first-cut hay (i.e., the first harvest of the growing season). Of these, 11 samples originated from intensively managed grassland (HI), and 7 samples from extensively managed grassland (HE).

**Table 1:** Classification of hay samples in intensively managed grassland (HI) and extensively managed grassland (HE).

No.	Type of hay	Cutting date	Conditioning	Drying
1	HE	NA	Small square bale	In the barn
2	HE	07.24	Round bale	In the barn
3	HI	06.24	Small square bale	In the barn
4	HI	05.24	Large square bale	In the barn
5	HI	06.24	Large square bale	In the barn
6	HE	06.24	Large square bale	In the barn
7	HE	07.24	Round bale	In the field
8	HE	06.24	Round bale	In the field
9	HE	06–07.24	Round bale	In the field
10	HI	05.24	Round bale	In the field
11	HI	07.24	Round bale	In the field
12	HI	06.23	Round bale	In the field
13	HI	05.24	Small square bale	In the barn
14	HI	06.23	Round bale	In the field
15	HI	05–06.24	Round bale	In the field
16	HI	06.24	Round bale	In the field
17	HI	05.24	Small square bale	In the field
18	HE	05.24	Small square bale	In the field

NA = not available.

HI samples were defined as grass hay, predominantly composed of ryegrass (*Lolium* spp.) under Swiss conditions. The forage was dried either in the field or in a barn using a drying system, achieving a dry matter (DM) content of ≥85% (Coenen & Vervuert, 2010; Daccord et al., 2027; Harris et al., 2017; Kamphues, 2013). Intensively managed grassland was further characterized by regular fertilization with slurry and a higher cutting frequency (4–5 cuts per year).

HE samples originated from extensively managed grassland and were composed of a more diverse range of plant species, including less intensively managed grasses such as *Arrhenatherum elatius* L., *Festuca pratensis* Huds., *Dactylis glomerata* L., and *Trisetum flavescens* L., as well as various herbaceous species (Jeangros & Thomet, 2004). Extensive management was defined by the absence of fertilization, a first cut only after flowering and 2–3 cuts per year.

Classification of hay as HI or HE was based on farmer-reported management practices. In addition, HI samples were visually identified as being predominantly composed of *Lolium* spp. The precise botanical composition of the hay samples was not further analyzed in this study.

### 2.2 Hay sample collection

Hay samples were collected between March 17 and April 21, 2025. A minimum of 1.5 kg was collected for each sample, weighed with a scale type ES-PS01 designed for hay nets. Each sample comprised material collected from at least three bales (small square, large square, or round bales; Table 1). Within each bale, subsamples were obtained from at least ten randomly selected locations, as described by Glauser (2007). The hay samples were then stored in 60-L polyethylene bags. The air was carefully expelled from each bag, which was then sealed tightly with a cable tie.

### 2.3 Dry matter, botanical and structural composition of hay samples

For the determination of dry matter content, 150 g of each hay sample were dried at 105 °C for 24 h and weighed before and after drying according to Naumann and Bassler (1997).

For each forage sample, 300 g were weighed using an EK4150 scale and then used for functional and structural composition analysis. Hay samples were manually sorted into three functional groups - grasses, legumes, and herbs. In a subsequent step, the same samples were further separated morphologically into stems, leaves, and inflorescences (structural composition), following the method described by Gustavsson (2011).

Each fraction was then weighed separately to determine the respective proportion in the sample.

### 2.4 Sensory analysis

Sensory analyses were performed one week after sample collection with a scoring system developed by collaborators of Agroscope, the Swiss centre for agricultural research, affiliated with the Swiss Federal Office for Agriculture (FOAG), (Table 2; Wyss & Strickler, 2011).

**Table 2:** Scoring system for sensory analysis according to Wyss and Strickler (2011).

Parameter	Findings		Points
Odor	Very pleasant hay smell, aromatic		5
	Pleasant hay smell, aromatic		3
	Faint or absent odor		1
	Slight musty smell, scorched odor		0
	Strong musty or putrid odor		−3
Color	Little alteration in color (greenish to brownish)		5
	Brownish to brown or pale color		3
	Dark brown or very pale color		1
	Dark brown to black or grayish (mold growth)		0
Texture	Rich in stems, ears very visible, rough and rigid to the touch		5
	Few hard stems, few visible ears, less hard to the touch		3
	Many hard stems, withered grasses, very hard to the touch		2
	No stems, only leafy mass, very soft to the touch		0
Contamination	None (no dust), especially no mold present		5
	Slight presence of dust		1
	Strong presence of dust or soil		0
	Mold, toxic plants		−15
**Evaluation**	**Result** Very good Good Average Bad	**Sensory score** 16–20 10–15 5–9 <5	

### 2.5 Measurement of dust release from different hay qualities

Respirable dust was measured using a standardized sampling system adapted according to the procedure described by Herholz et al. (2020). The experimental setup consisted of a sealed clear plastic box (volume: 40 L) with a perforated central platform placed on a fitness vibration plate (VibroShaper). The box, filled with 100 g of hay, was connected to an SDS011 dust sensor (Inovafit, Shandong Architecture University, China) via a plastic tube for air sampling. The VibroShaper was operated at intensity level 15 to agitate the hay. PM₂.₅ and PM₁₀ concentrations were continuously recorded inside the box over a 3 min measurement period using an SDS011 sensor. The sensor is based on a laser light-scattering principle, whereby airborne particles are drawn into the device, illuminated by a laser beam, and the intensity of scattered light is detected and converted into particle size and mass concentration (Budde et al., 2018). The measurement duration was defined based on preliminary measurements demonstrating a consistent decline in dust concentrations after 3 min, indicating that peak exposure conditions had passed. The sensor was connected to a microcomputer that logged and transmitted concentration data (µg/m³) to a CSV file and wirelessly forwarded them to a central minicomputer (System SmartHorse®, MUUTU AG).

For each hay sample, three independent measurements were performed. Between measurements, the box was emptied, thoroughly cleaned, and refilled with a new 100 g hay sample. Baseline conditions were confirmed prior to each measurement. For each hay sample, three subsamples were analyzed. During dust measurements, PM₁₀ concentrations exceeded the detection limit in some samples, resulting in incomplete data for this parameter. A very strong correlation was observed between PM₁₀ and PM₂.₅ in the available measurements (r = 0.995). Therefore, only PM₂.₅ was included in the subsequent analyses. The mean PM₂.₅ concentration was calculated over the 3-min measurement period for each subsample and subsequently used to determine the overall mean and standard deviation.

### 2.6 Bioaerosol sampling and laboratory analysis

**Fig. 1:**
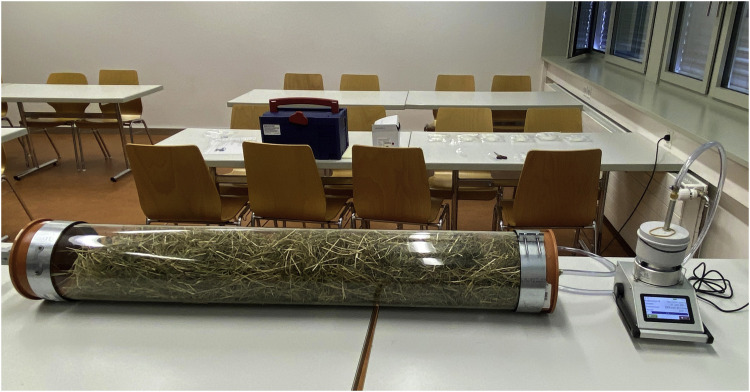
Microbial air sampling system (MBASS30). Legend: from left to right: cylinder containing 500 g of hay, connecting pipe, FA30 filter adapter, Microbial Air Sampling System (MBASS30).

Bioaerosol sampling was conducted to quantify airborne microbial concentrations (total aerobic, mesophilic bacteria, molds, yeasts, and actinomycetes) emitted from each hay sample. A Microbial Air Sampling System (MBASS30; Umweltanalytik Holbach GmbH, Germany) was used. For each measurement, 500 g of hay was placed in a 1-m PVC cylinder (40 L volume) with an inlet port (Fig. 1). The sampler operated at a flow rate of 30 L/min, drawing air through the cylinder and onto a sterile membrane filter. Each sampling cycle collected 300 L of air over 10 min. Prior to each measurement, room temperature and relative humidity were recorded. Indoor air (without hay samples) was collected before and after the measurement series using the Microbial Air Sampling System, and the corresponding plates were submitted to the laboratory for analysis. To correct for ambient background contamination, the mean values of the two blank measurements (pre- and post-study) were subtracted from each sample value (mean values: total aerobic, mesophilic bacteria, 45 CFU/m³; yeasts, 4 CFU/m³; molds, 12 CFU/m³; actinomycetes, 0 CFU/m³). Each sample was placed into the cleaned cylinder and the MBASS30 equipped with a new sterile filter was connected. A pre-sampling blank run using an old filter was performed to purge the system. A new sterile filter was then installed, and the sampler was operated at 30 L/min for 10 min. After sampling, the filter was removed, placed in a sterile container, and labeled with the sample ID. The cylinder was subsequently emptied, cleaned thoroughly, and the sampling room was ventilated for at least 10 min before the next run. All sample filters and two field blanks were stored refrigerated and submitted to the Laboratory of Molecular Analyses (PCR-Lab Services GbR, Germany) for microbial analysis. Two primer pairs were used to detect (i) aerobic mesophilic bacteria and actinomycetes and (ii) molds and yeasts. Primer sets targeted conserved regions of the 16S rDNA gene (bacteria/actinomycetes) and the 18S rDNA gene (molds/yeasts) (Table 3).

**Table 3:** Specific information for all primers used in the quantitative real-time PCR (qPCR)*.

Organism	Target gene	Designation	DNA sequence
Aerobic mesophilic bacteria	16S rDNA	forward primer alpha-F	5`-GCT CAG ATT GAA CGC TGG CGG-3`
	16S rDNA	reverse primer alpha-R	5`-ACT GCT GCC TCC CCC GTA GGA GT-3`
Actinomycetes	16S rDNA	forward primer A16F	5′- GGA TGA GCC CGC GGC CTA-3′
	16S rDNA	reverse primer A16R	5′-CCA GCC CCA CCT TCG AC-3′
Yeast	18S rDNA	forward primer Y18F	5′-TCC GTA GGT GAA CCT GCG G-3′
	18S rDNA	reverse primer Y18R	5′-T´CC TCC GCT TAT TGA TAT GC-3′
Moulds	18S rDNA	forward primer M18F	5`-GGT TCT ATT TTG GTT TCT A-3′
	18S rDNA	reverse primer M18R	5′-CTC TCA ATC TGT CAA TCC TTA TT-3′

⁎ Reference: G. Kaim, PCR-Lab Services GbR, Germany, unpublished data.

Genomic DNA was purified using the Exgene Cell SV Kit (GeneAll Biotechnology Co., Ltd., Seoul, South Korea) according to the manufacturer’s protocol. Quantitative real-time PCR (qPCR) was performed on a PTC Tempo Thermal Cycler (Bio-Rad, Hercules, CA, USA) using RealAmp 2× SYBR Green Master Mix (GeneAll, Seoul, South Korea). Cycling conditions consisted of an initial denaturation at 95 °C for 2 min, followed by 39 cycles of 95 °C for 10 s, 60 °C for 20 s, and 65 °C for 30 s. Melting curve analysis was conducted to confirm amplification specificity.

A positive qPCR signal indicated the presence of the respective microbial group; however, due to the use of conserved target regions, results represent total gene copy numbers and do not allow species-level identification or assessment of microbial viability.

Gene copy numbers were expressed per filter (300 L sampled air) and quantified using run-specific standard curves generated from serial dilutions of target rDNA sequences included in each run. This approach accounted for variability in amplification efficiency. Gene copy numbers were converted to colony-forming unit (CFU) equivalents based on the respective standard curves and expressed as CFU equivalents per m³ using a scaling factor of 1000/300.

### 2.7 Statistical analysis

Given the limited sample size (*n* = 18; 11 HI, 7 HE), analyses were conducted within an exploratory and descriptive framework without formal hypothesis testing. The objective of the statistical approach was to identify patterns and relationships among microbial, airborne dust (PM_2.5_), and botanical characteristics of the hay samples.

To account for the interrelated nature of the measured variables and to avoid multiple univariate comparisons, a principal component analysis (PCA) was performed on fifteen numerical variables, including microbial indicators (total aerobic, mesophilic bacteria, molds, yeasts, actinomycetes), dry matter, PM₂.₅ concentration, sensory traits (odor, color, structure, contamination level and overall score), as well as functional and structural characteristics (grasses, legumes, stems, inflorescences). All variables were centered and scaled prior to analysis (Jolliffe, 2002). The first principal components, explaining >70% of the total variance, were retained for subsequent analyses.

To explore grouping structures among samples, hierarchical cluster analysis was performed using the PCA scores combined with the binary variables hay type (HI vs. HE) and drying method (in the barn vs. in the field). A Gower distance matrix was used to account for the mixed data types (Gower, 1971), and clustering was conducted using Ward’s method (Ward, 1963). The optimal number of clusters was determined using the silhouette method (Rousseeuw, 1987).

Cluster assignments and multivariate patterns should be interpreted cautiously, as results may be sensitive to variable selection and the limited sample size. For interpretation, summary statistics of the original variables were calculated within each cluster.

Relationships among variables were further explored using pairwise plots and Spearman’s rank correlation coefficients, which are appropriate for small sample sizes and non-normal data. Correlation coefficients are presented as descriptive measures and may be sensitive to individual observations, particularly within small subgroups.

All analyses were performed in R 4.5.1 (R Core Team, 2025).

## 3 Results

### 3.1 Hay sample characteristics and sensory scores

Across all analyzed samples, grasses constituted the predominant functional fraction, with proportions ranging from 82.5% to 100%. Legumes were present only in minor proportions (0–4.2%). The highest proportion of legumes was observed in Sample No 3 (HI), whereas six samples (nos. 1, 2, 4, 12, and 18) contained no legumes. The proportion of other botanical components (herbs) ranged from 0% to 16.9%, with Sample No 6 (HE) exhibiting the highest share.

The dry matter (DM) content of the 18 hay samples ranged from 85.7% to 90.4% (Table 4). The proportion of stems varied between 40.8% and 83.7%, leaves between 12.4% and 58.2%, and inflorescences between 0.7% and 10.5%. In 17 of the 18 samples, stems represented the dominant structural fraction, exceeding both leaves and inflorescences. In contrast, Sample No 17 showed a reversed pattern, with a higher proportion of leaves than stems (58.2% vs. 40.8%). Across all samples, the proportion of inflorescences remained lower than that of both stems and leaves, with Sample No 13 (HI) exhibiting the highest inflorescence proportion (10.5%). Based on sensory evaluation, hay quality was classified as *very good* in four samples (scores 16–20), *good* in ten samples (scores 10–15), and *average* in four samples (scores 5–9).

**Table 4:** Hay type, dry matter (DM) content (%), structural composition (%), sensory evaluation scores, and the resulting overall hay quality classification.

No.	type of hay	DM (%)	Structural composition (%)			Sensory parameters (points)				Sensory score	Quality
			Stems	Leaves	Inflorescences	Odor	Color	Texture	Contamination		
1	HE	87.5	50.8	47.9	1.3	0	3	3	0	6	Average
2	HE	89.4	80.1	17.6	2.2	1	1	5	0	7	Average
3	HI	86.9	53.5	45.5	1.0	3	3	3	0	9	Average
4	HI	85.8	83.7	12.4	3.9	5	3	3	5	16	Very Good
5	HI	86.2	69.0	30.0	1.0	3	3	5	1	12	Good
6	HE	87.6	59.9	35.2	4.9	3	5	3	1	12	Good
7	HE	86.3	63.4	35.6	1.0	3	3	3	0	9	Average
8	HE	90.4	61.1	36.3	2.6	3	3	5	1	12	Good
9	HE	89.8	57.6	40.5	2.0	3	5	3	1	12	Good
10	HI	87.3	62.5	34.2	3.3	3	1	5	5	14	Good
11	HI	87.5	66.7	31.7	1.7	3	3	5	5	16	Very Good
12	HI	88.2	66.7	28.3	5.0	3	3	5	0	11	Good
13	HI	86.7	50.7	38.9	10.5	5	5	3	1	14	Good
14	HI	88.6	66.8	32.5	0.7	0	5	5	0	10	Good
15	HI	85.7	61.3	32.2	6.5	5	3	3	5	16	Very Good
16	HI	87.1	56.1	43.2	0.7	3	5	3	0	11	Good
17	HI	89.9	40.8	58.2	1.0	1	5	3	1	10	Good
18	HE	86.6	59.1	36.4	4.5	3	3	5	5	16	Very Good

HI = intensively managed grassland.

HE = extensively managed grassland.

### 3.2 Airborne dust and microbiological load

The raw data, including mean ± SD (µg/m³) for PM₂.₅ and microbial concentrations (CFU/m³), are presented in Table 5, providing an overview of particulate and microbial exposure associated with each hay type. Microbial concentrations (CFU/m³) were additionally expressed on a logarithmic scale (log₁₀) to facilitate comparison with results from other studies.

**Table 5:** Airborne dust PM_2.5_ (µg/m^3^) and microbiological contamination (CFU/m³) and expressed as log_10_ () in 18 hay samples.

**No.**	**Type of hay**	**Dust emissions PM_2.5_ (µg/m^3^)**		Airborne microbiological contamination (CFU/m³)			
		**Mean**	**SD**	**Total aerobic mesophilic bacteria**	**Molds**	**Yeasts**	**Actinomycetes**
1	HE	263.50	34.00	39’955 (4.6)	4’322 (3.6)	2’330 (3.4)	333 (2.5)
2	HE	130.65	7.86	59’955 (4.8)	5’655 (3.8)	2’997 (3.5)	667 (2.8)
3	HI	172.47	28.06	33’288 (4.5)	2’988 (3.5)	1’330 (3.1)	667 (2.8)
4	HI	25.95	5.00	29’955 (4.5)	2’322 (3.4)	1’330 (3.1)	667 (2.8)
5	HI	178.77	32.82	26’622 (4.4)	1’988 (3.3)	1’330 (3.1)	333 (2.5)
6	HE	179.04	36.78	49’955 (4.7)	5’322 (3.7)	2’330 (3.4)	667 (2.8)
7	HE	515.26	169.16	46’622 (4.7)	3’988 (3.6)	1’663 (3.2)	6’667 (3.8)
8	HE	363.84	74.18	39’955 (4.6)	3’322 (3.5)	1’330 (3.1)	333 (2.5)
9	HE	268.22	68.20	63’288 (4.8)	5’988 (3.8)	2’997 (3.5)	1’000 (3.0)
10	HI	123.80	53.86	56’622 (4.8)	5’322 (3.7)	2’330 (3.4)	1’000 (3.0)
11	HI	95.91	46.08	53’288 (4.7)	4’988 (3.7)	2’663 (3.4)	1’333 (3.1)
12	HI	122.79	31.67	26’622 (4.4)	2’322 (3.4)	997 (3.0)	333 (2.5)
13	HI	81.99	10.78	23’288 (4.4)	1’655 (3.2)	663 (2.8)	333 (2.5)
14	HI	104.40	28.64	43’288 (4.6)	3’988 (3.6)	1’663 (3.2)	667 (2.8)
15	HI	107.72	31.26	46’622 (4.7)	3’655 (3.6)	1’330 (3.1)	333 (2.5)
16	HI	475.08	338.54	56’622 (4.8)	4’988 (3.7)	2’330 (3.4)	1’000 (3.0)
17	HI	232.33	86.49	63’288 (4.8)	5’655 (3.8)	2’663 (3.4)	1’333 (3.1)
18	HE	91.91	12.84	66’622 (4.8)	5’988 (3.8)	2’663 (3.4)	1’000 (3.0)

HI = intensively managed grassland; HE = extensively managed grassland.

### 3.3 Cluster analysis

The PCA conducted on fifteen standardized numerical variables describing hay characteristics—including microbial load (four variables), dry matter, PM₂.₅ concentration, sensory traits (five variables), and structural and botanical traits (four variables)—indicated a strong reduction in dimensionality. The first four principal components explained 73% of the total variance and were retained for subsequent analyses. Hierarchical clustering based on the four PCA scores and two binary variables (hay type and drying method) suggested a four-cluster structure, supported by the silhouette method. Cluster quality was assessed using silhouette widths derived from the Gower distance matrix. Mean silhouette widths ranged from 0.29 to 0.45 across clusters, indicating moderate separation between clusters. Clusters 2 and 4 exhibited the highest mean silhouette widths (0.45 and 0.42, respectively), reflecting stronger internal cohesion, whereas Cluster 3 showed lower but still positive values (0.29), suggesting greater heterogeneity. Overall, silhouette analysis was consistent with a four-cluster solution, with predominantly positive silhouette values indicating reasonable agreement between individual samples and their assigned clusters.

**Fig. 2:**
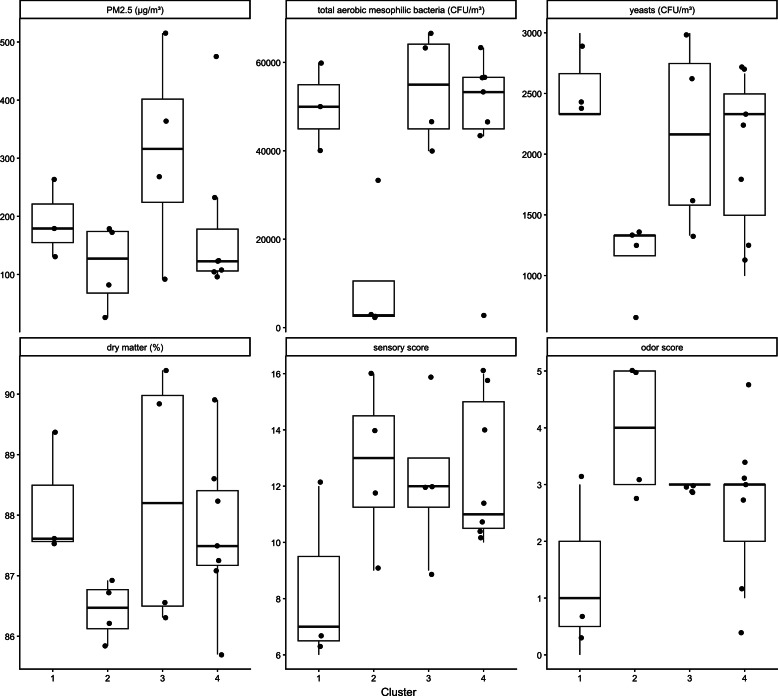
Characteristics of clusters 1–4 based on PM₂.₅ concentrations (µg/m^3^), total aerobic, mesophilic bacterial counts (CFU/m^3^), dry matter content (%), sensory and odor scores.

The clustering approach grouped the hay samples into the following sets: cluster 1 included hay samples 1, 2, and 6; cluster 2 included hay samples 3, 4, 5, and 13; cluster 3 included hay samples 7, 8, 9, and 18; and cluster 4 included hay samples 10, 11, 12, and 14–17 . The distribution of samples across clusters according to hay type and drying method is shown in Table 6. Mean germ counts (CFU/m³ and as log_10_), PM_2.5_ (µg/m³) dust values, sensory parameters and score, and dry matter content (%) were assigned to clusters 1–4 (Fig. 2; Table 7).

**Table 6:** Assignment of hay type or drying method to clusters 1–4.

Type of Hay	Cluster 1	Cluster 2	Cluster 3	Cluster 4
HI	0	4	0	7
HE	3	0	4	0
				
**Drying method** In the Field	0	0	4	7
In the barn	3	4	0	0

HI = intensively managed grassland.

HE = extensively managed grassland.

**Table 7:** Sensory and botanical evaluation, mean PM₂.₅ dust levels (µg/m^3^), and mean airborne microbial counts (CFU/m³) in hay clusters 1–4.

Cluster	Total aerobic mesophilic bacteria	Yeasts	Molds	Actino-mycetes	Dust PM_2.5_
**1**	49,955 (4.7)	2552 (3.4)	5100 (3.7)	556 (2.7)	191
**2**	10,288 (4.0)	1163 (3.1)	2238 (3.3)	500 (2.7)	115
**3**	54,122 (4.7)	2163 (3.3)	4822 (3.7)	2250 (3.4)	310
**4**	46,050 (4.7)	1997 (3.3)	4417 (3.6)	857 (2.9)	180
**Cluster**	**Sensory score**	**Odor**	**Color**	**Structure**	**Contamin-ation**
**1**	8.3	1.3	3.0	3.7	0.3
**2**	12.8	4.0	3.5	3.5	1.8
**3**	12.2	3.0	3.5	4.0	1.8
**4**	12.6	2.6	3.6	4.1	2.3
**Cluster**	**Dry Matter (%)**	**Grasses** **(%)**	**Legumes** **(%)**	**Stems** **(%)**	**Inflorescences (%)**
**1**	88.2	93.2	0.2	63.6	2.8
**2**	86.4	94.9	1.3	64.2	4.1
**3**	88.3	95.6	0.6	60.3	2.5
**4**	87.7	97.1	0.8	60.1	2.7

Hay from intensively managed grassland (HI) was clearly separated from hay from extensively managed grassland (HE). In addition, field-dried samples were distinct from barn-dried samples. Descriptive statistics of the original variables calculated within each cluster indicated coherent and distinct cluster profiles. The clusters showed differences in microbial counts, dry matter content, particulate matter concentrations, sensory scores, and functional composition. Clusters 1 and 3 tended to exhibit higher mean germ counts and dust levels, higher dry matter content, and lower sensory scores; all samples in these clusters were HE (Fig. 2; Table 7). In contrast, Cluster 2 was characterized by lower mean germ and dust contamination and the lowest dry matter content. This cluster also included hay with the highest average odor scores and comprised exclusively HI that had been barn-dried (Fig. 2; Table 7). These cluster patterns should be interpreted as descriptive groupings rather than definitive classifications.

**Fig. 3:**
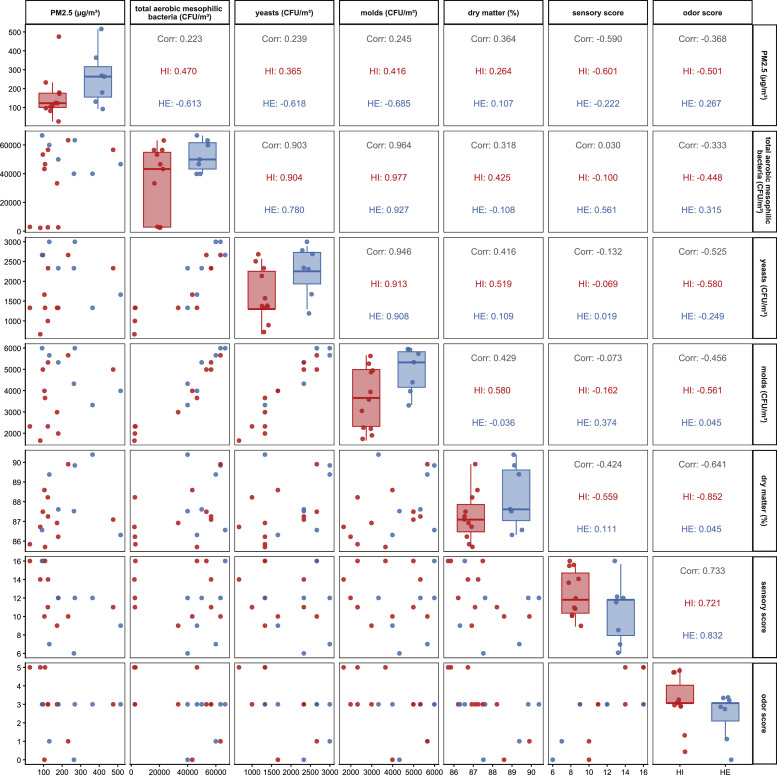
Pairwise relationships among particulate matter PM_2.5_ (µg/m^3^) and microbial variables (CFU/m^3^), sensory score, odor score and dry matter (%) by hay type HI and HE. **Legend:** HI = samples from intensively managed grassland. HE = samples from extensively managed grassland.

**Fig. 4:**
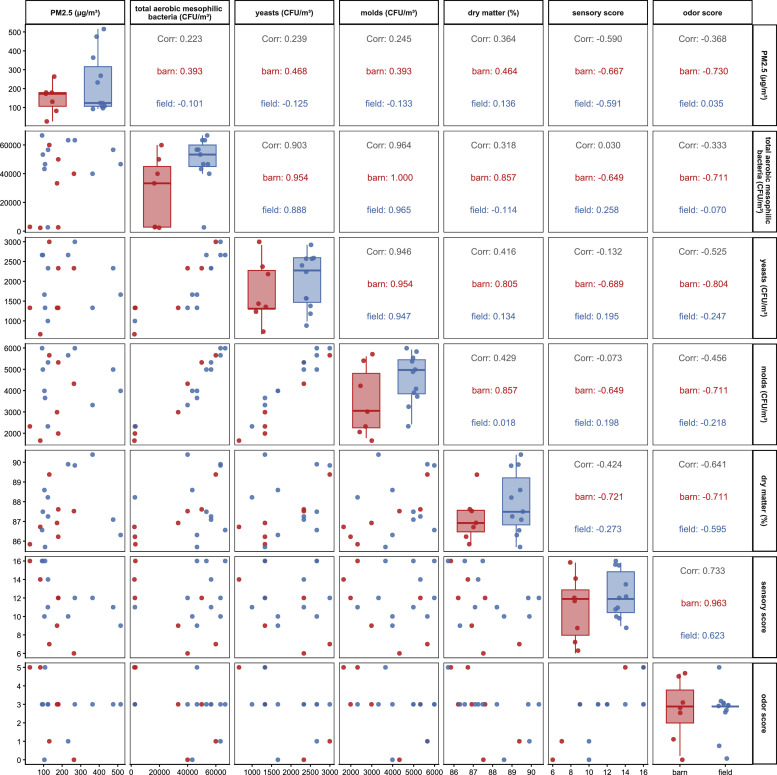
Pairwise relationships among particulate matter PM_2.5_ (µg/m^3^) and microbial variables (CFU/m^3^), sensory score, odor score and dry matter (%) by drying method (field or barn). **Legend:** Barn = samples from barn-dried hay. Field = samples from field-dried hay.

Pairwise correlations were calculated using Spearman’s rank correlation coefficient for HI versus HE (Fig. 3) and for field-dried versus barn-dried hay (Fig. 4). Differences in the relationships among PM₂.₅, microbial variables, and quality parameters were observed according to drying method and management intensity.

Positive correlations between PM₂.₅ and total aerobic, mesophilic bacterial counts were observed in barn-dried hay (*ρ* = 0.393) and in intensively managed hay (*ρ* = 0.470). In contrast, correlations appeared weak or negative in field-dried hay (*ρ* = −0.101) and extensively managed hay (*ρ* = −0.613).

Spearman’s rank correlation coefficient suggested a negative association between PM₂.₅ and odor score in barn-dried hay (*ρ* = −0.730), compared with a coefficient of *ρ* = 0.035 in field-dried hay. Given the small subgroup sizes, these associations should be interpreted as indicative rather than conclusive.

Overall, PCA, hierarchical clustering, and silhouette diagnostics suggested a consistent multivariate structure in the data. Despite the limited sample size, the combined approach identified cluster patterns broadly aligned with hay type and drying method, while preserving meaningful variation in microbial, physical, and functional and structural characteristics. These results provide an exploratory but robust multivariate framework for characterizing forage samples under differing production conditions.

## 4 Discussion

The aim of the present study was to assess the quantity of dust and microorganisms emitted into the air by hay of different qualities. Equine asthma is influenced by a wide range of environmental and management-related risk factors, as comprehensively reviewed by Mańkowska and Witkowska (2024) and the release of dust and microorganisms, in particular in the breathing zone of horses, represents a major factor in the pathogenesis of equine asthma (Bouverat et al., 2025a; Mańkowska & Witkowska, 2024). Among these, exposure to dust and airborne microorganisms originating from hay is considered particularly relevant (Vandenput et al., 1997). In this context, the implementation of standardized measurement approaches that integrate airborne dust and microbial concentrations, combined with a clustering of hay quality according to exposure potential, represents a promising advancement for future risk assessment frameworks.

Although no validated threshold values for dust or microbial contamination in stable environments or in the breathing zones of horses are currently available, systematic and standardized measurements remain highly informative. Such data provide a quantitative basis for characterizing exposure and identifying potential risk patterns. Drawing on established methodologies for environmental and food safety risk assessment, including the framework proposed by Hassan et al. (2025) and Serhan et al. (2024), these approaches could support the development of targeted prevention strategies tailored to equine respiratory health.

Hay samples originated from different farms in Switzerland and were from the first cut. According to sensory evaluation, none of the hay samples were of insufficient quality, which is a prerequisite for feeding horses. The release of dust and microorganisms into the air from hay of different qualities was measured using standardized methods to examine the effect of hay quality itself. Under stable conditions, exposure levels may vary considerably depending on factors such as horse behavior, stable ventilation, and the method of hay feeding (e.g., loose on the floor, in nets, or in automatic feeders) (Bouverat et al., 2025a; Fritz, 2017; Garlipp et al., 2010; Harris et al., 2017; Berndt et al., 2010; Herholz et al., 2020a, b; Ivester et al., 2012). The World Health Organization (WHO) guideline values for maximum mean daily dust exposure in humans are 25 µg/m³ for PM₂.₅ and 50 µg/m³ for PM₁₀. In the present study, mean PM₂.₅ concentrations exceeded the recommended WHO limits in all hay samples (Table 5). Only hay sample 4 (HI, barn-dried) showed values close to the threshold, with a PM₂.₅ concentration of 25.95 µg/m³.

In this study, PM₂.₅ was measured in a small experimental chamber under standardized and continuously agitated conditions. These measurements do not reflect the exposure levels that horses or humans would experience under normal stable conditions. However, the controlled setup is appropriate for comparative assessment of different hay samples. Accordingly, the results allow the conclusion that hay samples generating lower PM₂.₅ mass concentrations under these standardized conditions would be expected to result in lower potential exposure for both horses and humans.

Nevertheless, the concentrations measured in the experimental chamber are not representative of the larger air volume within a stall or barn. Therefore, they cannot be directly extrapolated to real-life exposure scenarios. Measurements within the breathing zone would be required to draw robust conclusions regarding actual exposure levels and to evaluate them in relation to WHO threshold values.

Hay sample 4 was part of cluster 2, together with samples 1, 2, and 6. All samples in this cluster originated from intensively managed grassland and were barn-dried.

These hay samples were associated with the lowest PM_2.5_ exposure values, the lowest total aerobic, mesophilic bacterial counts, and the best average odor score. This is in accordance with the study of Bouverat et al. (2025b), which suggested that sensory examination, particularly assessment of abnormal odor, may be indicative of dust exposure. The study by Garlipp et al. (2010) evaluated an air-driven particle separation technology applied to various materials, including hay. Dust concentrations, including PM₂.₅, were measured using a TEOM 1400a device. For untreated hay, PM₂.₅ concentrations of up to 2500 µg/m³ were reported. However, these results are not directly comparable to those of the present study, as a larger amount of hay (5 kg) was analyzed using a different methodological approach.

In another study, Wolny-Koładka (2018) assessed PM_2.5_ concentrations in the air of three stables using a DustTrak monitoring device, reporting values ranging from 49 to 362 µg/m³. The PM_2.5_ values obtained in the present study generally fell within this range, with one hay sample (no. 4) below and three samples (nos. 7, 8, and 16) above this interval.

A direct comparison between the findings of Wolny-Koładka (2018) and those of the present study is limited by substantial differences in experimental design and methodological approach. Wolny-Koładka (2018) quantified airborne PM₂.₅ concentrations in stable environments using a real-time DustTrak aerosol monitor, whereas the present study assessed particulate matter associated with hay samples under controlled, sample-specific conditions. These fundamentally different matrices—ambient stable air versus feed material—differ in particle generation and dispersion dynamics and therefore in measured concentrations. Taken together, these differences in experimental design highlight the strong influence of methodological factors on absolute PM concentrations and underline the importance of interpreting dust measurements in a strictly study-specific context.

In the present study, cluster 2 (HI, barn-dried) was associated with relatively low PM₂.₅ concentrations and low total aerobic, mesophilic bacterial counts. In contrast, clusters 1 and 3 exhibited relatively low PM₂.₅ concentrations but high total aerobic, mesophilic bacterial counts (Fig. 2). This finding is consistent with the study by Séguin et al. (2010a), which reported no relevant correlation between total airborne dust and CFU levels, indicating that hay contamination by mold CFUs cannot be inferred from dustiness alone.

Microbial and biochemical changes in self-heating hay, depending on its water content, can lead to marked increases in thermophilic actinomycetes and fungi. These microorganisms are known to induce airway sensitization in humans (farmer’s lung) and animals (Lacey & Lacey, 1964; Vandenput et al., 1997). During hay handling, concentrations of up to 1.6 × 10⁹ spores per m³ of air have been recorded in farm buildings, with actinomycete spores accounting for up to 98% of total airborne spores (Lacey & Lacey, 1964). Dutkiewicz et al. (1994) reported airborne concentrations of thermophilic actinomycetes in stables for Arabian racehorses ranging from 1000 to 209,600 CFU/m³.

In the present study, the highest airborne concentration of actinomycetes was 6667 CFU/m³ (sample no 7), with a mean concentration of 2250 CFU/m³ in hay samples from cluster 3 (HE, field-dried). In six samples (nos. 9, 10, 11, 16, 17, and 18), airborne actinomycete concentrations ranged from 1000 to 1333 CFU/m³; these samples included both HE and HI hay types but were exclusively field-dried. The remaining samples showed comparatively low concentrations (333–667 CFU/m³), irrespective of drying method. As only total airborne actinomycetes were quantified, the proportion of thermophilic actinomycetes was likely lower than that reported by Dutkiewicz et al. (1994).

The data presented here were obtained under standardized experimental conditions and should therefore be interpreted as controlled reference values rather than direct representations of field exposure. In stable environments, airborne microbial loads are shaped by a complex interplay of factors, including animal activity, feeding management, ventilation, and barn microclimate. Consequently, the concentrations reported in the present study provide a comparative experimental framework, while actual exposure levels under practical conditions are likely to vary considerably.

Furthermore, key environmental parameters such as storage temperature, relative humidity, ventilation conditions, and storage duration were not systematically recorded. These factors are known to substantially influence microbial growth and dust generation in stored hay. Their omission may have contributed to the variability observed in microbial and dust measurements and limits a comprehensive interpretation of the differences between samples.

From a statistical perspective, the study is limited by the relatively small sample size, which restricts inferential conclusions. The applied multivariate analyses are therefore exploratory and descriptive in nature. Observed clustering patterns and correlations provide indications of potential relationships but require confirmation in a prospective adequately powered study.

### 4.1 *Effects of hay from intensively* vs. *extensively managed grasslands on hygienic quality*

The observed separation between HI and HE hay may be partly attributed to differences in functional composition and management intensity. Intensively managed grasslands dominated by *Lolium* spp. typically produce more uniform swards with higher biomass yield, which may influence drying dynamics and subsequently microbial development. In contrast, extensively managed grasslands with higher floristic diversity often result in lower biomass yield and include species with varying stem structures and moisture contents, potentially leading to more heterogeneous drying conditions. Such differences may affect microbial colonization and survival during haymaking.

Similar effects of floristic composition on forage hygienic quality have been reported by Séguin et al. (2010b), indicating that plant diversity and management intensity may influence microbial characteristics in hay. In the present study, cluster 2 showed the lowest mean airborne mold counts (3.3 log₁₀ CFU/m³), whereas clusters 1 and 4 (both HE) exhibited higher values (3.7 log₁₀ CFU/m³). Séguin et al. (2010b) reported lower dust and mold contamination in *Lolium*-dominated swards compared with more diverse mixtures, with mean airborne mold counts of 5.6 log₁₀ CFU/m³ for *Lolium perenne* hay, 3.2 log₁₀ CFU/m³ for Swiss commercial hay, and 6.2 log₁₀ CFU/m³ for multi-species hay.

Although comparable air-sampling approaches were used, their culture-based methodology differs from the qPCR-based quantification applied in the present study, limiting direct comparability. Nevertheless, these findings provide a general indication of the magnitude of airborne microbial concentrations.

A key limitation of the present study is that botanical composition was not determined at the species level. Consequently, the contribution of individual plant species cannot be assessed, and any comparison with species-specific findings from the literature remains speculative. The observed differences are therefore more likely to reflect general effects of sward structure and management intensity rather than specific botanical composition.

### 4.2 *Effects of field* vs. *barn drying of hays on hygienic quality*

The results showed a clear distinction between field-and barn-dried hay samples. Regarding hygienic quality, this difference was most pronounced in cluster 2 (HI, barn-dried) and less evident in cluster 4 (HE, barn-dried). Séguin et al. (2010a) investigated the impact of agricultural practices and production processes on forage hygienic quality, defined as all airborne dust constituents potentially involved in the etiology of equine asthma, including respirable dust, molds, pollen, and mycotoxins. In their study, early-cut, barn-dried hay from intensively managed grasslands released 29,000 CFU/m³ of total aerobic, mesophilic bacteria, whereas field-dried samples reached 475,000 CFU/m³ at 25 °C. In comparison, the present study recorded mean total aerobic, mesophilic bacterial concentrations of 10,288 CFU/m³ for cluster 2 (HI, barn-dried) and 46,050 CFU/m³ for cluster 4 (HI, field-dried). Both studies therefore demonstrate a clear difference in hygienic quality between field-dried and barn-dried hay. However, aerobic, mesophilic bacterial counts in HI field-dried samples were substantially lower in the present study, which may be attributable to differences in botanical composition, analytical methodology, and/or weather conditions during field drying. Séguin et al. (2011) further showed that barn drying had little effect on airborne particle concentrations (0.3–20 µm) but reduced fungal contamination. They also reported that hay harvested at lower dry matter content (75%vs. 85%) exhibited higher respirable dust and fungal loads. In the present study, field versus barn drying did not have a clear effect on PM₂.₅ concentrations, which were similar in cluster 4 (field-dried, HI) and cluster 1 (barn-dried, HE), suggesting variability within drying methods and a stronger influence of grass composition (HI vs. HE). Importantly, the combination of HI production and barn drying (cluster 2) resulted in markedly improved hygienic quality, with lower dust and microbial loads than all other clusters (Table 7).

## 5 Conclusions

The present study demonstrates that both hay production type (HI vs. HE) and drying method (field-dried vs. barn-dried) are key determinants of dust and microorganism release into the air. These findings highlight the importance of forage processing and handling practices for airborne exposure relevant to equine respiratory health. Future research should focus on generating standardized and methodologically comparable datasets across different forage types, drying methods, and storage conditions to enable the establishment of reliable threshold values for health risk assessment. In addition, studies under real stable conditions, including direct monitoring of the equine breathing zone, are needed to better reflect actual exposure scenarios. Furthermore, the inclusion of roughage from later harvests and of insufficient sensory quality is recommended, as this represented a limitation of the present study.

## Data availability statement

The data that support the findings of this study were not deposited in an official repository and are available from the corresponding author upon request.

## Ethical statement

This study did not involve live animals, animal experimentation, or animal handling. The research was conducted exclusively on hay samples collected for laboratory analysis. Therefore, according to institutional and national guidelines, ethical approval from an animal care and use committee was not required.

All procedures complied with relevant institutional and national regulations.

## Author agreement

Herewith we certify that all authors have seen and approved the revised version of the manuscript being submitted. They warrant that the article is the author’s original work, hasn't received prior publication and isn't under consideration for publication elsewhere.

## CRediT authorship contribution statement

**Jan Kocher:** Writing – review & editing, Writing – original draft, Project administration, Investigation, Data curation. **Christelle Mossu:** Writing – review & editing, Writing – original draft, Project administration, Investigation, Data curation. **Jasmin Wandel:** Writing – review & editing, Writing – original draft, Formal analysis. **Georg Kaim:** Writing – original draft, Data curation. **Beat Reidy:** Writing – review & editing, Writing – original draft. **Conny Herholz:** Writing – review & editing, Writing – original draft, Supervision, Methodology, Conceptualization.

## Declaration of competing interest

The authors declare that they have no known competing financial interests or personal relationships that could have appeared to influence the work reported in this paper.
